# Rabacfosadine for naïve canine intermediate to large cell lymphoma: Efficacy and adverse event profile across three prospective clinical trials

**DOI:** 10.1111/vco.12605

**Published:** 2020-05-08

**Authors:** Corey F. Saba, Craig Clifford, Kristine Burgess, Brenda Phillips, David Vail, Zachary Wright, Katie Curran, Timothy Fan, Robyn Elmslie, Gerald Post, Douglas Thamm

**Affiliations:** ^1^ College of Veterinary Medicine, Department of Small Animal Medicine & Surgery University of Georgia Athens Georgia USA; ^2^ Hope Veterinary Specialists Malvern Pennsylvania USA; ^3^ Department of Clinical Sciences Cummings School of Veterinary Medicine at Tufts University North Grafton Massachusetts USA; ^4^ Veterinary Specialty Hospital of San Diego San Diego California USA; ^5^ School of Veterinary Medicine, Department of Medical Sciences University of Wisconsin‐Madison Madison Wisconsin USA; ^6^ VCA Animal Diagnostic Clinic Dallas Texas USA; ^7^ Carlson College of Veterinary Medicine, Department of Clinical Sciences Oregon State University Corvallis Oregon USA; ^8^ College of Veterinary Medicine, Department of Veterinary Clinical Medicine University of Illinois at Urbana‐Champaign Urbana Illinois USA; ^9^ VRCC Veterinary Specialty & Emergency Hospital Englewood Colorado USA; ^10^ Chief Medical Officer FidoCure Palo Alto CA; ^11^ Colorado State University Flint Animal Cancer Centre Fort Collins Colorado USA

**Keywords:** canine, chemotherapy, lymphoma, rabacfosadine, treatment naive

## Abstract

While current lymphoma therapies induce remission in most dogs, drug‐resistant relapse is common, creating a need for novel agents. Rabacfosadine (RAB), a double prodrug of the acyclic nucleotide phosphonate 9‐(2‐phosphonylmethoxyethel) guanine (PMEG), preferentially targets lymphoma cells with reduced systemic toxicity compared with PMEG. Previous studies evaluating RAB administered every 21 days have suggested efficacy in both naïve and relapsed subjects; however, no large studies of RAB as a single agent have been reported in previously untreated dogs with intermediate to large cell lymphoma. The purpose of this study was to evaluate the safety and efficacy of RAB in dogs with previously untreated (excluding corticosteroids) lymphoma. Sixty‐three dogs received up to five RAB treatments every 21 days (16 at 0.82 mg/kg and 47 at 1.0 mg/kg) as a 30 minutes intravenous infusion, with (n = 23) or without (n = 40) concurrent corticosteroids. Response assessment and adverse event (Ae) evaluation were performed every 21 days via Veterinary Cooperative Oncology Group (VCOG) criteria. The overall response rate was 87% (52% CR, 35% PR). The overall median progression free interval was 122 days (199 for CR, 89 for PR and 153 days for all responders). T‐cell immunophenotype and corticosteroid pre‐treatment were predictive of inferior outcomes on multivariate analysis. AEs were most commonly of gastrointestinal origin (hyporexia/diarrhoea) and generally resolved with supportive treatment and/or dosage adjustment. Three dogs experienced VCOG‐CTCAE grade 5 delayed pulmonary fibrosis. In conclusion, RAB administered every 3 weeks is generally well tolerated and demonstrates substantial antitumour activity in dogs with previously untreated intermediate to large cell lymphoma.

## 1 INTRODUCTION

Lymphoma, specifically diffuse large cell B‐cell lymphoma and peripheral T‐cell lymphoma (PTL), is one of the most common cancers in dogs.^1^, ^2^ The current standard of care treatments consist of multi‐agent chemotherapy regimens. Most commonly, oncologists rely on doxorubicin (DOX)‐based protocols [eg, a cyclophosphamide, hydroxydaunorubicin (doxorubicin), Oncovin (vincristine), prednisone (CHOP)‐based protocol]. With this approach, multiple drugs are given weekly to biweekly, generally over the course of 19 to 26 weeks.^1^, ^3^, ^4^, ^5^ The frequency of veterinary visits associated with this intensive treatment protocol can become burdensome for some owners and is impossible for others. Less intensive protocols, where cytotoxic drugs are given every 3 weeks, offer a less stringent treatment option, but with the potential risk of shorter remission durations and survival times^6^, ^7^, ^8^; however, for many owners, this is a reasonable compromise.

Rabacfosadine (RAB; Tanovea‐CA1; also referred to as VDC‐1101 or GS‐9219) is a double prodrug of the acyclic nucleotide phosphonate 9‐(2‐phosphonylmethoxyethel) guanine (PMEG), that preferentially targets lymphoma cells with reduced systemic toxicity compared with PMEG.^9^ It was conditionally approved by the U.S. Food and Drug Administration for the treatment of canine lymphoma in 2016. According to its label, it may be given every 21 days for up to five treatments to dogs with lymphoma of any type. Studies evaluating RAB in both treatment‐naive and relapsed or treatment‐refractory dogs with lymphoma have reported overall response rates of approximately 50% to 100%, with higher response rates and longer response durations observed in dogs with B‐cell lymphoma and those that are less heavily pre‐treated.^9^, ^10^, ^11^, ^12^

For the most part, RAB has been well tolerated, typically associated with low grade or mild adverse events (AEs) including neutropenia and gastrointestinal (GI) signs. The dermatologic AE associated with RAB often manifests as pruritic otitis externa and/or erythemic skin lesions on the dorsum and in the inguinal areas. These dermatological AEs typically resolve with supportive therapy and/or dose reductions and/or delays.^9^, ^10^, ^11^, ^13^, ^14^ Additionally, pulmonary fibrosis is a reported toxicity associated with RAB administration. Although reported in approximately 4% of RAB‐treated dogs, it is potentially life‐threatening. The mechanism of this AE is not understood, but it appears to be idiosyncratic. Careful monitoring with thoracic radiographs for evidence of pulmonary pathology is encouraged.^9^, ^10^, ^11^, ^13^, ^14^

The purpose of this report was to describe the safety and efficacy of RAB in client‐owned dogs with intermediate to large cell lymphoma naïve to any prior treatment. Data were extracted from three separate prospective multi‐institutional studies (VC‐003, VC‐007 and VC‐010), which were conducted between 2011 and 2017. The findings of this report are intended to serve as a more informative guide for oncologists using RAB in the setting of treatment‐naïve, intermediate to large cell, canine lymphoma.

## 2 METHODS

### 2.1 Inclusion/exclusion criteria

Data on client‐owned dogs with treatment naïve intermediate to large cell lymphoma that were treated with RAB across three separate prospective clinical trials (VC‐003, VC‐007 and VC‐010) were extracted. For inclusion in each of these trials, dogs were required to have a cytologic or histologic diagnosis of multicentric, intermediate to large cell lymphoma. Confirmation of immunophenotype using immunohistochemistry, immunocytochemistry, flow cytometry or polymerase chain reaction for antigen receptor rearrangement (PARR) was strongly recommended but not required. Similarly, post‐mortem examination of dogs that died while on study was encouraged but not required. Only data from dogs with treatment‐naïve lymphoma were extracted, with the caveat that dogs receiving prior corticosteroids were considered treatment naïve. Information regarding the type, duration of treatment, and dose of corticosteroids was not readily available, rather this variable was recorded as “yes” prior treatment with corticosteroids or “no” prior treatment with corticosteroids.

Screening tests included physical examination, complete blood count (CBC), serum biochemical profile and urinalysis to ensure dogs met inclusion criteria. Thoracic radiographs were strongly recommended. Adequate bone marrow and organ function, defined as absolute neutrophil count ≥2000 cells/uL, haematocrit ≥25%, platelet count ≥75 000 cells/uL, creatinine ≤2.5 mg/dL, total bilirubin ≤ the upper limit of normal (ULN), ALT ≤3 times ULN or if >3 times ULN, serum bile acids ≤ULN and a modified ECOG performance score ≤1 were required for inclusion.^8^ West Highland white terrier dogs and/or dogs with pulmonary pathology possibly predisposing to fibrosis were excluded. Study protocol approval was obtained from Institutional Animal Care and Use Committees and/or Clinical Review Boards according to individual institutional requirements. Other treatment options, including no treatment, were discussed with all owners. Owners were allowed to make their own educated decisions about the appropriate choice for their pet, and signed informed consent was obtained from all owners prior to study entry.

### 2.2 Trial design

RAB was provided by VetDC, Inc. (Fort Collins, Colorado). Depending upon the study (VC‐003, VC‐007 and VC‐010), dogs were treated at 0.82 or 1.0 mg/kg. RAB was reconstituted and diluted with sodium chloride for injection, USP to achieve a total infusion volume of 2 mL/kg and was administered intravenously (IV) over 30 minutes. Treatments were repeated every 21 days for up to five total treatments. The treatment schedules were uniform across all studies and are outlined in Table 1.

**TABLE 1:** Study schedule

Day	RAB treatment	PE	LN evaluation	CBC	Serum chemistry	UA	Thoracic radiographs
Pre‐enrolment (day −7 to −1)				X	X	X	X^a^
Day 0	X	X	X	X^b^	X^b^	X^b^	
Day 7		X		X			
Day 21	X	X	X	X	X^c^	X^c^	
Day 28^d^		X		X			
Day 42	X	X	X	X	X	X	
Day 63	X	X	X	X	X		
Day 84	X	X	X	X	X	X	X^a^
Monthly rechecks		X	X				Every other month

Abbreviations: CBC, complete blood count; LN, lymph node; PE, physical examination; RAB, rabacfosadine; UA, urinalysis.

a Thoracic radiographs were strongly recommended but not required.

b If CBC, serum chemistry and urinalysis were performed and evaluated within 7 days of day 0, these were not repeated on day 0.

c These were not required for all studies.

d This visit was only required in dogs experiencing a dose‐limiting toxicity following the first treatment.

Treatment response was based on measurements (using callipers) of peripheral target lesions using the Veterinary Cooperative Oncology Group (VCOG) Response Evaluation Criteria for Peripheral Nodal Lymphoma.^15^ Dogs experiencing complete response (CR) received a total of five RAB treatments; thereafter, monthly rechecks were performed until progressive disease (PD) was noted. Dogs experiencing partial response (PR) or stable disease (SD) after five treatment cycles were considered off‐study upon completion of the fifth treatment cycle. Once PD was noted, dogs were removed from the study and were eligible to pursue other treatment(s) as deemed appropriate by the attending oncologist and at the owner's discretion.

### 2.3 AE assessment

Clinical, haematological and biochemical AEs were assessed based on patient history provided by the owner, physical examination and blood work as outlined in Table TABLE 1. AEs were graded according to the Veterinary Cooperative Oncology Group Common Terminology Criteria for Adverse Events (VCOG‐CTCAE) v1.1.^16^ Dose‐limiting toxicities (DLTs) were defined as any grade 3 or 4 non‐hematologic toxicity, any uncomplicated (eg, no fever and bleeding) grade 4 hematologic toxicity, or any complicated grade 3 or 4 hematologic toxicity. Dermatological lesions deemed less than grade 3 according to VCOG‐CTCAE v1.1^16^ criteria but considered clinically substantial and/or extensive enough to warrant protocol alteration were considered DLTs. Dose reductions and/or delays of up to 2 weeks were permissible to manage AEs. If a DLT was observed, the dose was reduced by up to 20% for future RAB administrations. Treatment of AEs was undertaken at the discretion of the attending clinician.

### 2.4 Statistical analysis

Continuous data were expressed as median and range, and categorical data as frequencies and percentages. The objective response rate (ORR) and progression‐free interval (PFI) were the primary efficacy endpoints. The ORR was defined as the percentage of evaluable patients experiencing CR or PR as their best response. The PFI was calculated from the date of treatment initiation to the date of PD. Dogs were censored if they were lost to follow‐up prior to documentation of PD, if they were withdrawn for a reason other than PD, or if they died of a confirmed cause unrelated to RAB treatment or lymphoma before PD development. Continuous variables were compared between groups of patients using a two‐tailed, unpaired T test or Mann‐Whitney test depending on data normality, which was assessed using a D'Agostino Pearson omnibus test. Categorical variables were compared between cohorts using a two‐tailed Fisher's exact test. The Kaplan‐Meier method was used to estimate and display the distribution of PFI. Differences between potential prognostic subsets were compared using logrank analysis. Variables with a univariate *P* value of <.15 were incorporated into a forward stepwise logistic regression multivariable Cox proportional‐hazards model to compare the multiple variables for effect on PFI. Variables with values of *P* ≤ .5 were considered significant. All statistical analysis was performed with the commercial software packages (Prism v.8, GraphPad Software, La Jolla, California; SPSS v.25, IBM, Armonk, New York).

### 2.5 Cell line validation statement

No cell lines were used in this study.

## 3 RESULTS

Data from 63 dogs were abstracted. Studies were conducted across 11 study sites including Colorado State University, Hope Veterinary Specialists, Oregon State University, Tufts University, University of Georgia, University of Illinois, University of Wisconsin‐Madison, VCA Animal Diagnostic Clinic, Veterinary Cancer Centre, Veterinary Referral Centre of Colorado and Veterinary Specialty Hospital of San Diego. Twenty‐nine different breeds were represented with the most common breeds including mixed breed dogs, Golden Retrievers, Labrador Retrievers and Boxers. Other patient characteristics including sex, age, body weight, prior treatment with corticosteroids, concurrent corticosteroids, RAB dose received and immunophenotype are summarized in Table 2.

**TABLE 2:** Patient characteristics

Sex	Spayed female	24
	Intact female	5
	Neutered male	31
	Intact male	3
Median age		7 years (range: 2–15 years)
Median body weight		30.1 kg (5.4‐78 kg)
Prior treatment with corticosteroids	Yes	15
	No	48
Concurrent corticosteroids	Yes	40
	No	23
Dose received	0.82 mg/kg	16
	1.0 mg/kg	46
	Other	1
Immunophenotype	B‐cell	44
	T‐cell	15
	Not known	4

Three dogs were withdrawn from the study prior to the first response assessment. The ORR for all evaluable dogs (n = 60) was 87%. Best responses were CR in 52% (31 dogs), PR in 35% (21 dogs), SD in 5% (3 dogs), and PD in 8% (5 dogs). The median time to first response was 21 days, and median time to maximal response was 42 days.

Dogs with B‐cell lymphoma were significantly more likely to respond and to have CRto RAB treatment as compared with dogs with T‐cell lymphoma (*P* < .0001 for both ORR and CR percentage). The ORR for the 42 evaluable dogs with B cell lymphoma was 97%, which included 62% CR (26 dogs) and 35% PR (15 dogs). One dog had PD at the time of first response evaluation. The ORR for the 14 evaluable dogs with T‐cell lymphoma was 50%, which included 22% CR (3 dogs), 28% PR (4 dogs) and 22% SD (3 dogs). Four dogs (28%) had PD at the time of first response evaluation. Of the dogs where immunophenotype was unknown, there were two each of CR and PR. Other factors that were assessed for effect on response included patient demographic factors (age, body weight, sex), substage of disease, RAB dose administered and pre‐treatment or co‐treatment with corticosteroids. None had an impact on response rate.

**FIGURE 1:**
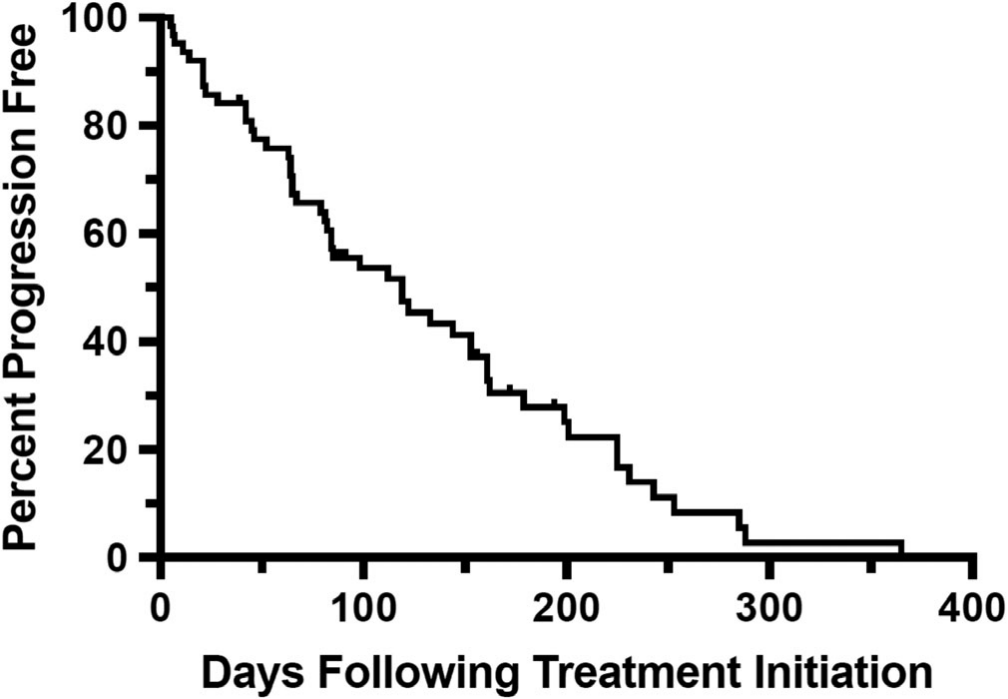
Progression free interval for all dogs

**FIGURE 2:**
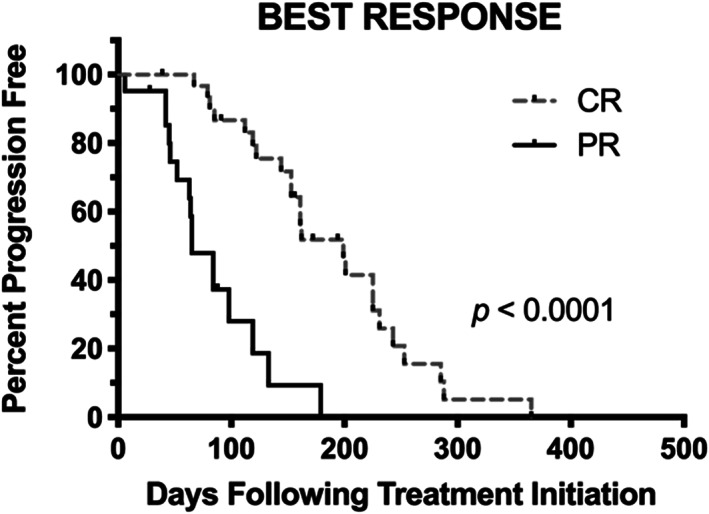
Response progression free interval

Thirteen dogs were censored from PFI analysis. Five dogs were lost to follow‐up prior to progression, six were withdrawn because of owner non‐compliance or AEs, one was euthanized in remission because of a gall bladder mucocele and one was euthanized in remission because of diagnosis of a second tumour (hemangiosarcoma). The median follow‐up time for all censored patients was 91 days (range: 28‐194 days). The overall median PFI was 122 days (range: 5‐365 days; Figure 1). The median PFI for all responders (CR + PR) was 153 days (range: 6‐365 days). Dogs experiencing CR had a significantly longer PFI as compared with dogs experiencing PR (199 days; range: 67‐365 days vs 89 days; range: 6‐179 days; *P* < .0001; Figure 2).

On univariate analysis, significant negative predictors of PFI included lack of response to treatment, T‐cell immunophenotype, prior treatment with corticosteroids and concurrent treatment with corticosteroids. Insignificant variables included approximate stage and substage of disease and RAB dosage received. Response to treatment (*P* < .001), T‐cell immunophenotype and prior treatment with corticosteroids remained significant on multivariable analysis. When response was eliminated as a variable, T‐cell immunophenotype (*P* < .001) and prior treatment with corticosteroids (*P* = .019) retained significance.

The AE profile was comparable to previous studies, with GI AEs most common. The percentage of dogs experiencing hematologic, GI and unique (dermatopathy and pulmonary) AEs are summarized in Table 3, with AEs divided by dosage received (0.82 vs 1.0 mg/kg) in each table. Hyporexia of any grade was the only AE that was significantly more common in the 1.0 mg/kg dosage group (*P* = .04). One dog received 0.82 mg/kg initially and was escalated to 1.0 mg/kg; therefore, this dog was not included in this analysis; however, it did not experience any AEs at either dosage. Pulmonary fibrosis was confirmed with histopathology in only one dog with grade 5 pulmonary fibrosis; the others were suspected based on clinical signs. Aside from grade 5 pulmonary fibrosis, AEs were self‐limiting and resolved with supportive care and/or dosage modification. Only four dogs required dose reductions or delays.

**TABLE 3:** Hematologic, gastrointestinal, dermatologic and pulmonary adverse events (AEs) at 0.82 mg/kg (A) and 1.0 mg/kg (B)

AE/grade	1	2	3	4	5
(A) Occurrences and highest grade severity of AEs (0.82 mg/kg); n=16					
Neutropenia	13%				
Thrombocytopenia	6%				
Anaemia	19%				
Vomiting	25%	6%			
Diarrhoea	25%	13%			
Hematochezia					
Hyporexia	19%		6%		
Weight loss	19%	13%			
Dermatopathy	25%				
Pulmonary fibrosis					6%
(B) Occurrences and highest grade severity of AEs (1.0 mg/kg); n = 46					
Neutropenia	7%				
Thrombocytopenia	7%				
Anaemia	13%				
Vomiting	30%	4%			
Diarrhoea	24%	15%	4%		
Hematochezia	4%	2%			
Hyporexia	43%	11%	4%		
Weight loss	11%	20%	13%		
Dermatopathy	26%	20%	2%		
Pulmonary fibrosis	2%				4%

Abbreviation: AE, adverse event.

## 4 DISCUSSION

The results of this study provide evidence that RAB is an effective treatment for dogs with treatment‐naïve, multicentric, intermediate to large cell lymphoma, with an 87% overall response rate and a median PFI of 122 days. Dogs with B‐cell lymphoma were more likely to respond and had a longer PFI than dogs with T‐cell lymphoma. Dogs previously treated with corticosteroids had a significantly shorter PFI than those without (81 vs 144 days).

AEs were similar to those previously reported, including myelosuppression, GI effects, dermatopathy and pulmonary fibrosis;^9^, ^10^, ^11^, ^13^, ^14^ only hyporexia was significantly more common at 1.0 mg/kg as compared to 0.82 mg/kg. Three dogs developed clinical and radiographic signs consistent with grade 5 pulmonary fibrosis; otherwise, AEs were self‐limiting and resolved with supportive care and/or dosage modification.

The current standard of care treatment for canine lymphoma is a multi‐agent chemotherapy protocol (eg, a CHOP‐based protocol or in some cases, an alkylator‐rich protocol) with cytotoxic drugs given weekly to biweekly, generally over the course of 19 to 26 weeks.^1^, ^3^, ^4^, ^5^ While feasible for some clients, the frequency of veterinary visits associated with this intensive treatment protocol is demanding, and the treatment is costly. For some, a reasonable compromise is a less intensive protocol, where cytotoxic drugs are given every 3 weeks. DOX has been evaluated as a first‐line single agent protocol. Several investigators have reported on the efficacy of DOX in the treatment of canine lymphoma. Reported ORRs, CR rates and median response durations have been 74% to 87%, 52% to 78%, and 80.5 to 169 days, respectively.^6^, ^7^, ^17^, ^18^, ^19^, ^20^ Lomustine and a tapering dose of corticosteroids have also been retrospectively evaluated as first‐line treatment in 17 dogs with lymphoma. The reported ORR was 53%, with a median duration of 39.5 days; these results lead the authors to conclude that lomustine should not be used as a first‐line therapy for canine lymphoma.^8^ Here, we report an ORR of 87% and a median PFI of 122 days for all RAB treated dogs (B‐ and T‐cell). Dogs with B‐cell lymphoma had a higher ORR of 97%. It is impossible to compare the results of the current study to those from other independent studies; however, it is noteworthy that our results appear to be within the range of those reported for single agent DOX treatment.

Despite the acceptable results observed with single‐agent DOX, all of the aforementioned studies concluded that these single agent treatment protocols are not intended to replace the current standard of care; rather, they simply provide a less time consuming and potentially less costly treatment option.^6^, ^7^, ^8^ That being said, drug resistance remains an inevitable consequence of the treatment of canine lymphoma, even when multi‐agent protocols are used.^1^, ^3^, ^4^, ^5^, ^21^ The unique mechanism of action of RAB^9^ makes it an attractive, novel agent that may be combined with other chemotherapy agents and/or incorporated into CHOP‐based protocols in attempt to extend the time to lymphoma relapse and prolong ultimate drug resistance. The results reported here could justify evaluating this type of combinatorial treatment, in that they demonstrate the efficacy of RAB in the treatment of naïve, intermediate to large cell canine lymphoma. Furthermore, aside from the typical AEs of myelosuppression and GI effects, RAB does not have overlapping unique toxicities (eg, cardiotoxicity, peripheral neuropathy, sterile haemorrhagic cystitis) associated with other drugs in the CHOP protocol.^1^, ^3^, ^4^, ^5^, ^9^, ^10^, ^22^ Another treatment consideration is the combination of RAB and DOX, alternated every 21 days. This particular protocol was evaluated in 54 dogs with previously untreated lymphoma; the ORR was 84%, with a median PFI of 194 days,^13^ and may support this an attractive, convenient multi‐agent protocol where the cytotoxic drugs are administered at less frequent intervals and fewer treatment visits relative to CHOP‐based protocols.

The finding that T‐cell immunophenotype and prior treatment with corticosteroids are negative prognostic factors is not surprising. It is doubtful that these are unique to RAB, as prior studies have shown that the ORRs, response durations, and overall survival times are significantly shorter in dogs with large cell T‐cell lymphoma^1^, ^23^, ^24^, ^25^ and in dogs previously treated with corticosteroids.^1^, ^26^, ^27^ It is noteworthy however, that half of the dogs with T‐cell lymphoma responded to RAB, supporting the notion that RAB should not be discounted as a possible treatment option for dogs with T‐cell disease. Admittedly, RAB is not a p‐glycoprotein (P‐GP) substrate, so upregulation of the multidrug resistance gene and P‐GP induction cannot explain the finding that pre‐treatment with corticosteroids was associated with an inferior prognosis. We can speculate that it is probable that corticosteroids induce drug resistance via mechanisms other than P‐GP induction. For example, corticosteroid resistance in human leukaemia can be associated with enhanced Toll‐like receptor stimulation, resulting in enhanced JAK‐STAT and PI3K signalling and epigenetic de‐repression of expression of the anti‐apoptotic molecule BIM, all of which could theoretically confer cross‐resistance to a variety of antineoplastic agents.^28^ That said, we do not know why prior corticosteroid treatment was a negative prognostic factor.

When dogs developed signs of dermatopathy or otitis, these AEs were assumed to be RAB related, even if concurrent infection was detected. We did this so as not to under‐estimate how often this AE occurs. Admittedly, as a result, we may be over‐attributing RAB as the cause. Nonetheless, the dermatologic AEs seen here are consistent with those previously reported with RAB treatment. The mechanism of this AE remains unclear. Fortunately, especially when caught early, most dermatological AEs are mild and self‐limiting. The knowledge that dermatopathies occur with RAB treatment is important and should prompt clinicians to closely monitor dogs' skin and ears during RAB treatment. If noted, it is best to address these AEs early, as they can be successfully managed with dose reductions and/or delays and supportive medications, rather than discontinuation of RAB.^9^, ^10^, ^11^, ^13^, ^14^, ^29^

Pulmonary fibrosis, another AE of RAB, occurred in these studies, at a similar frequency to studies reported previously.^6^, ^9^ The mechanism of this remains unknown. The timing of pulmonary fibrosis, including grade 5 pulmonary fibrosis, is also unclear.^9^, ^10^, ^11^, ^13^ Grade 5 pulmonary fibrosis occurred in three dogs in these studies at 119, 133 and 144 days from the start of treatment. However, only one of these dogs underwent necropsy examination. Nonetheless, although pulmonary fibrosis is an infrequent complication of RAB treatment, the authors strongly encourage regular thoracic imaging in treated dogs.

Interestingly, both dermatologic and pulmonary toxicity occur in human patients treated with bleomycin. Dermatologic AEs including erythema, hyperpigmentation and vesicle formation reportedly occur in approximately 50% of treated patients; this is thought to be related to cumulative dose. Potentially life‐threatening pulmonary toxicity occurs in up to 10% of treated patients. While the mechanism of bleomycin‐related pulmonary toxicity is not entirely understood, oxidative lung damage, relative deficiencies of the deactivating enzyme bleomycin hydrolase in tissues such as the lung, genetic susceptibility and inflammatory cytokines are thought to be involved. Potential risk factors in people include concurrent administration of other pulmonary toxic drugs, thoracic irradiation, inhalation of high oxygen concentrations, older patient age and possibly concomitant use of granulocyte colony stimulating factor (G‐CSF).^30^, ^31^ While it is possible that similar mechanisms may explain the cause of RAB pulmonary fibrosis, additional studies are needed to thoroughly investigate the cause and time of this AE in RAB‐treated dogs.

A potential limitation of the current report is that, although these studies were conducted prospectively, the data were extracted retrospectively. Information regarding the type, duration of treatment and dose of corticosteroids was not readily available, making it difficult to comment further on this significant variable. It is also impossible to say whether or not some of the short‐lived responses were simply because of corticosteroid administration; however, concurrent treatment with corticosteroids did not alter the likelihood of response statistically. One could argue that PARR should not be used as a sole test to determine immunophenotype. Therefore, another limitation could be under‐ or over‐reporting of B vs T cell lymphoma. With that being said, the majority of these cases were immunophenotyped with assays other than PARR. As with previous RAB studies, owners were not asked to keep daily dairies at home to prospectively record any potential AEs, so subtle or mild constitutional and GI AEs (eg, lethargy, hyporexia, vomiting, diarrhoea) may have been under‐reported. Additionally, target lesion measurement and grading of AEs were performed by a team of clinicians, not necessarily the same clinician at each visit, possibly introducing bias. However, by using the VCOG Response Evaluation Criteria for Peripheral Nodal Lymphoma criteria and the CTCAE, which allow for inter‐individual measurement differences and provide specific criteria for AE grading, respectively, we suspect the bias was limited. Finally, necropsies were not required making it difficult to definitively assess causes of death in patients on study.

In conclusion, RAB appears to be an effective treatment for dogs with treatment‐naïve, multicentric, intermediate to large cell lymphoma, with an 87% overall response rate and a median PFI of 122 days. The response rate and PFI were longer in dogs with B cell lymphoma; however, half of the dogs with T cell lymphoma also responded to treatment. Continued careful monitoring for the unique dermatologic and pulmonary AEs is warranted in dogs receiving RAB. These results provide guidance for oncologists using RAB in the setting of treatment‐naïve, intermediate to large cell canine lymphoma and may justify the inclusion of RAB in the more aggressive multi‐agent CHOP‐based protocols and/or in combination with other single agent lymphoma treatment protocols.

## CONFLICT OF INTEREST

Dr Burgess, Phillips, Post, Saba, Thamm, and Vail are members of the VetDC Clinical Advisory Board. Dr Thamm is a shareholder in VetDC.

## Data Availability

Data Availability Statement: The data that support the findings of this study are available from the corresponding author upon reasonable request.
